# Microglial TLR4 is Critical for Neuronal Injury and Cognitive Dysfunction in Subarachnoid Hemorrhage

**DOI:** 10.1007/s12028-022-01552-w

**Published:** 2022-07-01

**Authors:** Rezwanul Islam, Frank Vrionis, Khalid A. Hanafy

**Affiliations:** 1 Department of Biomedical Sciences, Charles E. Schmidt College of Medicine, Florida Atlantic University, Boca Raton, FL USA; 2 Marcus Neuroscience Institute, Boca Raton Medical Center, Boca Raton, FL USA

**Keywords:** Subarachnoid hemorrhage, Microglia, Toll-like receptor, Neuronal injury, Cognitive dysfunction

## Abstract

**Background:**

Toll-like receptor 4 (TLR4) activation causes excessive production of proinflammatory mediators and an increased expression of costimulatory molecules that leads to neuroinflammation after subarachnoid hemorrhage (SAH). Although TLR4-mediated inflammatory pathways have long been studied in neuroinflammation, the specific glia implicated in initiation and propagation of neuroinflammation in SAH have not been well elucidated. In this study, we investigated the involvement of glial TLR4 including microglia and astrocytes in brain damage and poor neurological outcome.

**Methods:**

In this study, global TLR4 knockout, cell-specific TLR4 knockout, and floxxed control male and female mice were used. The mice were injected with 60 μl autologous blood near the mesencephalon to induce SAH; animals were euthanized on postoperative day 7 for immunohistochemistry of glia and apoptotic cells. Microglial morphology was evaluated by using immunofluorescence density quantification to determine correlations between morphology and neuroinflammation. Microglial depletion was accomplished with the intracerebroventricular administration of clodronate liposomes. Cognitive function was assessed with Barnes maze.

**Results:**

On postoperative day 7 after SAH induction, neuronal apoptosis was markedly reduced in the clodronate liposome group compared with phosphate-buffered saline control liposomes, and cognitive performance in the clodronate group was improved, as well. Differences in microglial activation, assessed by morphometric analysis, and neuronal apoptosis were significantly greater in wildtype knockouts compared with cell-specific and global TLR4 knockouts. The mice lacking TLR4 on astrocytes and neurons showed no differences compared with wildtype mice on any end points.

**Conclusions:**

Our data suggest that microglial depletion with the intracerebroventricular administration of clodronate can improve the cognitive function in an SAH mouse model, and TLR4 is critical for microglial activation and neuronal injury. Only microglial TLR4 is necessary for brain damage and poor cognitive outcome rather than astrocyte or neuronal TLR4. Thus, microglial TLR4 could be a potent therapeutic target to treat SAH-associated neuronal injury and protect against cognitive dysfunction.

## Introduction

Approximately 30,000 Americans will have an aneurysmal subarachnoid hemorrhage (SAH) this year, and approximately one third of the survivors will have some form of cognitive dysfunction [1, 2]. Vasospasm—long thought to be the mechanism of neuronal damage—when effectively treated, showed no improvement in cognitive outcome [3–6]. Although the moniker has now changed to delayed cerebral injury (DCI), the mechanism leading to DCI are poorly understood.

Neuroinflammation in post-SAH neuronal damage may be involved, given that heme and methemoglobin can act as toll-like receptor 4 (TLR4) agonists, as our lab and others have shown in hemorrhagic stroke [7–9]. Although TLR4 has a role in post-SAH inflammation [10, 11], the connection to cognitive dysfunction and the specific glia involved in post-SAH neuronal injury have yet to be determined. Our lab and others have previously shown that microglia have a critical role in mediating neuronal injury after SAH [12], but conditional knockouts of TLR4 on individual glial populations have never been performed. We believe further elucidation of specific glial TLR4 involvement is critical to develop strategies aimed at decreasing microglial-mediated neuroinflammation after SAH.

In our study, we tried to elucidate the roles of neuronal, astrocyte, and microglial TLR4 in the neuronal injury and cognitive function after SAH. Our data showed microglial activation, neuronal apoptosis, and cognitive dysfunction in the presence of TLR4, whereas, the lack of microglial TLR4 was sufficient to prevent any cognitive dysfunction or neuronal injury after SAH. On the other hand, astrocyte and neuronal TLR4 were not necessary to mediate any of the SAH-induced injury based on our results from mice lacking astrocyte and neuronal TLR4. Thus, our data suggest that microglial activation is critical to the pathogenesis of SAH-induced cerebral injury and that microglial TLR4, but not astrocyte or neuronal TLR4, is necessary for this post-SAH cerebral injury. Therefore, microglial TLR4 might be a potential target for injury after hemorrhagic stroke.

## Materials and Methods

### Animals

All animal experiments detailed herein complied with the regulations formulated by the Institutional Animal Care and Use Committee at Florida Atlantic University. All surgical manipulations were performed under general anesthesia with a 10:4 mg/kg mixture of ketamine (VetOne) and xylazine (Akorn Animal Health), respectively. For procedures, 8–12 week-old male and female mice, all of a C57BL/6 background, were obtained from the Jackson Laboratory, unless otherwise indicated: B6.129P2(Cg)-Cx3Cr1^tm2.1(cre/ERT2)Litt^ (Cx3Cr1^CreER^, stock number 021160-a tamoxifen-inducible myeloid-driven Cre promoter), B6.Cg-Tg(Nes-cre)1Kln (Nestin^Cre^, stock no. 003771-an early astrocyte and neuron-driven Cre promoter), B6(Cg)-TLR4^tm1.1Karp^ (TLR4^fl/fl^, courtesy of Dr. Tim Billiar), B6(Cg)-TLR4^tm1.2Karp^ (TLR4^−/−^, stock number 029015), and B6.SJL-Ptprc (C57BL/6 CD45.1 + mice, stock number 002014). The following murine crosses were generated: Cx3Cr1^CreER^:TLR4^fl/fl^ (tamoxifen-inducible to create a microglial specific knockout of TLR4) and Nes^Cre^: TLR4^fl/fl^ (a neuron and astrocyte-specific knockout of the TLR4). All mice received 4 mg of a 0.02 mg/µL subcutaneous injection of tamoxifen 1 month before any experimentation. The research personnel who performed the surgical procedures were different from the personnel who performed cognitive assays, ensuring appropriate blinding [13].

### SAH

The method used to induce SAH has been previously tested and validated in a mouse model [5, 12]. After the mice were anesthetized with intraperitoneal administration of xylazine (4 mg/kg) and ketamine (100 mg/kg), SAH was performed as previously described by our lab using a standard stereotaxic instrument set-up (Kopf Instruments, Tujunga, CA) [14]. To open the skin overlying the anterior skull, a slightly off-midline incision was performed. Then, a burr hole was drilled into the anterior skull, 4.5 mm anterior to the bregma. A total of 60 µl nonheparinized ventricular blood collected from another C57BL/6 wildtype (WT) donor mouse was injected over a 10-s period with a 27-gauge spinal needle (BD spinal needle, reference number 405081) at a 40° caudal angle 3 mm deep into the drilled burr hole. The needle was left in place for 5 min to prevent backflow of blood.

### Intracerebroventricular Administration

Mice were anesthetized as described above. Two burr holes were drilled 0.22 mm posterior to the bregma, 1 mm lateral, and 2.25 mm in depth to enter the bilateral ventricles. Pulled glass capillaries were used to inject 8 μl clodronate (a generous gift provided by Professor Reto Schwendener) [15] or phosphate-buffered saline (PBS) liposomes were divided equally between the ventricles for more than 12.5 min. The capillaries were held in place for 2.5 min thereafter to prevent any regurgitation, followed by skin closure. Intracerebroventricular (ICV) injections were performed on postoperative days (PODs) 1 to 2 after the SAH procedure. WT mice were randomly assigned to the following four treatment groups equally: WT SAH sham + ICV normal saline, WT SAH + ICV PBS (SAH + PBS), and WT SAH + ICV clodronate (Clod) (SAH + Clod). Lab personnel performing surgical procedures were not the same as those performing cognitive assays to allow for appropriate blinding.

### Immunohistochemistry and Terminal Deoxynucleotidyl Transferase dUTP Nick end Labeling Staining

Frozen brains were fixed and then were cut into 9-μm serial coronal sections with a Leica CM3050 S cryostat. Sections were postfixed with 2% Paraformaldehyde for 10 min and permeabilized with 0.5% Triton X-100/PBS for 10 min. Slides were then blocked in 10% goat serum in PBS for 60 min at room temperature [14]. Primary antibodies for Tmem-119, CD45, GFAP (Cell signaling, St. Louis, MO), TLR4 (Santa Cruz Biotechnology, Santa Cruz, CA), or β-III Tubulin (Millipore, Cambridge, MA) were applied at a dilution of 1:500, followed by secondary incubation with Alexa Fluor antibodies at a dilution of 1:500. Fluorescent microscopy was done on a Zeiss Axio Scope (Carl Zeiss Inc., Thornwood, NY). Brightness and contrast of images were adjusted in Image J software (National Institutes of Health). For terminal deoxynucleotidyl transferase dUTP nick end labeling (TUNEL) staining, slides were fixed in 4% Paraformaldehyde for 20 min and permeabilized with 0.1% Triton X-100 for 2 min. After washing with PBS, sections were covered with enzyme and label solution (In Situ Cell Death Detection Kit, TMR red; Roche Life Science) for 1 h at 37 °C in a humidified atmosphere. Lab personnel interpreting TUNEL stains were not aware of the groups to which they were assigned.

### Immunofluorescence Density and Nuclear Quantification

To achieve appropriate consistency in cell quantification, three images were obtained for each area of interest to quantify positive cells. Quantification was performed using ImageJ, the raw count of all microglia nuclei (Tmem-119 + /DAPI +) within a 500 × 500-pixel region of interest centered within the greater 1024 × 1024-pixel field. In addition, within the same region of interest, the raw count of the activated microglia nuclei (Tmem-119 + /DAPI +) and damaged neuron nuclei (β-III Tubulin + /TUNEL +) were determined. Microglial immunofluorescence (IF) density quantification was performed by measuring the green signal using the ImageJ (National Institutes of Health) software.

### Barnes Maze for Spatial Learning Evaluation

Cognitive function and spatial learning were tested on the Barnes maze as described previously, with minor modifications [14]. In brief, the paradigm consisted of a white circular platform with a diameter of 90 cm. Along the perimeter of the platform, 20 equal-sized holes were located. One hole led to a box to from which the animals could escape the open platform. Cardboard placards with different visual cues were placed around the platform. The location of the escape box and the visual cues were kept constant during the initial acquisition period. Prior to SAH, the mice underwent spatial acquisition for 7 days, with three trials per animal per day. Animals were allowed to explore the maze for 3 min. Once they entered the escape box, they were allowed to remain in the box for 1 min. The intertrial interval was 15 min for each animal, with extensive cleaning of the platform after each animal to eliminate olfactory cues. Animals that did not enter the escape box within 3 min were guided to the correct hole. After 7 days of acquisition, SAH was performed as described above. Spatial memory testing, which consisted of one trial per animal per day, was started on day 1 after SAH and continued for 7 days. To test for flexibility and relearning, the location of the goal box was moved 180° from its original position on day 4 (spatial reversal), while visual cues were kept at the initial position. Maze procedures were performed by an investigator blinded to the genotype and treatment groups.

### Statistical Analysis

Sample size was determined based on our work in the Journal of Clinical Investigation (2015) using the mean and common standard deviation of the latency time in the Barnes maze. Multiple experimental groups were compared using repeated-measures two-way analysis of variance (ANOVA) with Bonferroni’s post hoc test for in vivo TUNEL and Tmem-119 staining of multiple brain regions, and the results are presented as the mean ± the standard error of the mean (GraphPad Prism). Differences were considered significant at *P* < 0.05. No animals were excluded from these results. No significant difference was observed between sexes, so equal numbers of male and female mice were combined to form each group. Confounders were minimized by performing surgery at the same time of the day to eliminate circadian variation. Further, sham and SAH surgeries were performed together in a form of permuted block randomization.

## Results

### Clodronate Administration Effective for Neuroprotection and Cognitive Improvement After SAH in Mice

**Fig. 1:**
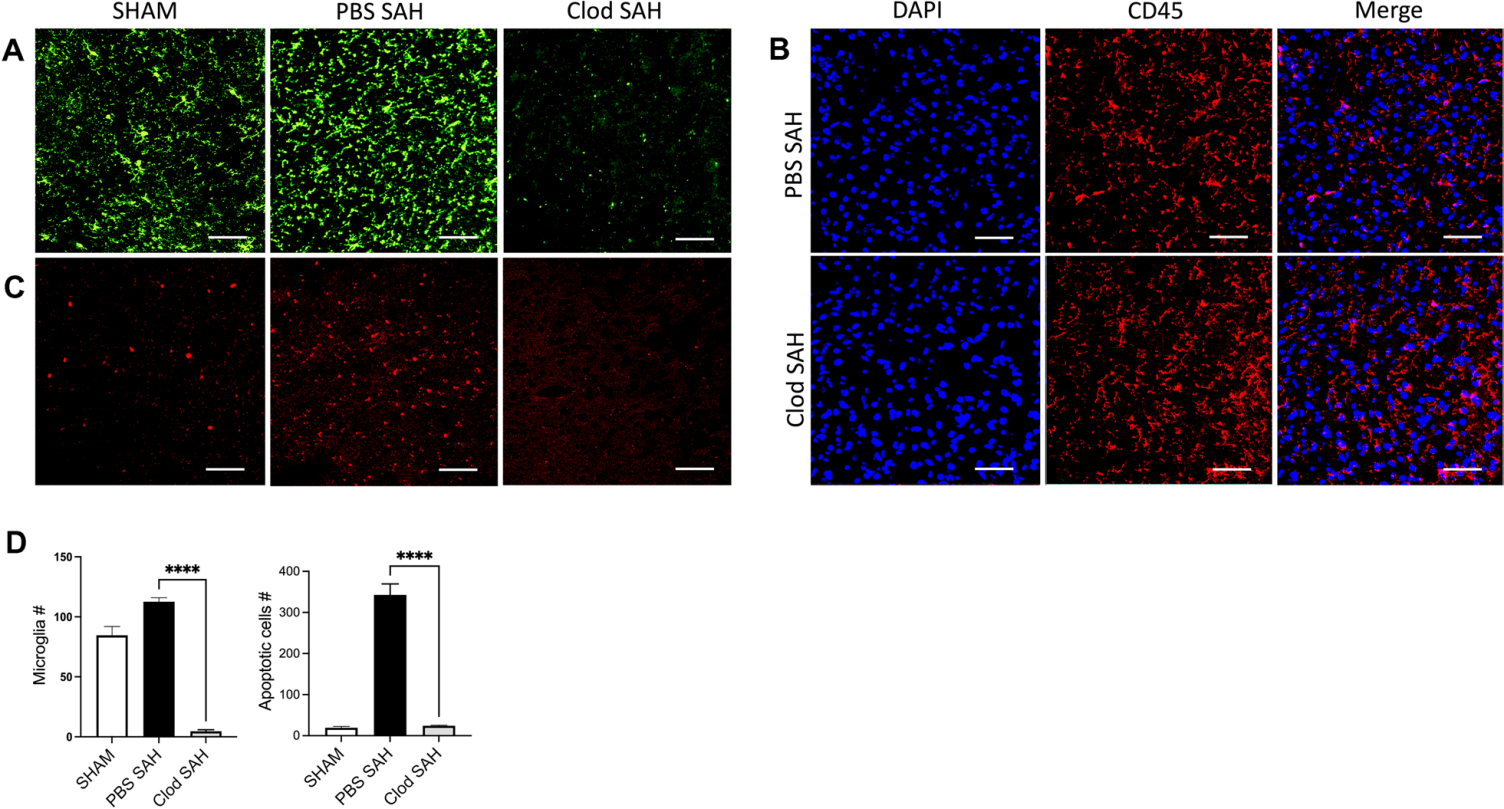
Microglial depletion by clodronate injection. Tmem119 (**a**) and CD45 (**b**) staining in wild type SAH on POD 7 after ICV administration of PBS liposomes and clodronate liposomes. **c** TUNEL staining in wild type SAH on POD 7 after ICV PBS and clodronate administration. **d** Quantification of microglia and apoptotic neurons on POD 7 in both PBS and clodronate treated groups. *n* = 6 for each group and all bar graphs represent mean ± SEM. One-way ANOVA showed significant differences with *P* < 0.05. Scale bars: 10 μm in all panels. *ANOVA* analysis of variance, *Clod* clodronate, *DAPI* 4′,6-diamidino-2-phenylindole, *ICV* intracerebroventricular, *PBS* phosphate-buffered saline, *POD* postoperative day, *SAH* subarachnoid hemorrhage, *SEM* standard error of the mean, *TUNEL* terminal deoxynucleotidyl transferase dUTP nick end labeling

**Fig. 2:**
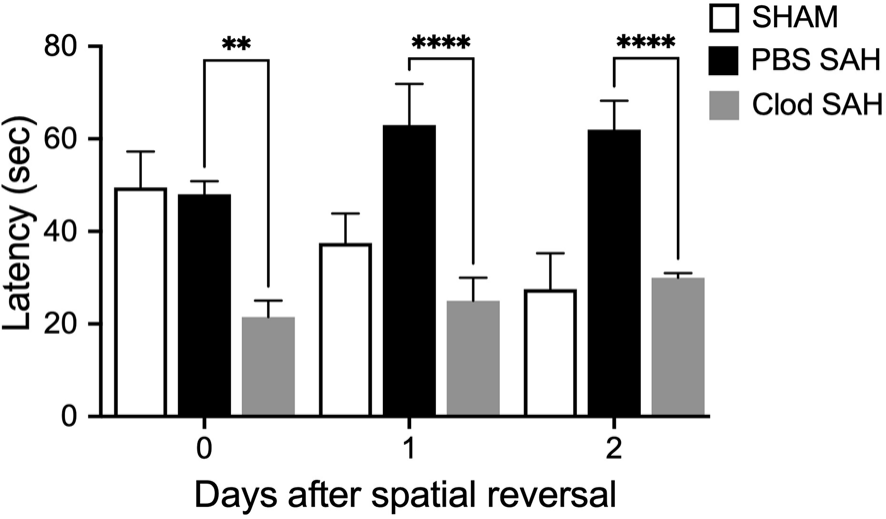
Cognitive function test. Total latency in seconds for spatial memory function testing by Barnes maze test for sham, SAH + PBS, and SAH + Clod. *n* = 6 for each group, and all bar graphs represent mean ± SEM. One-way ANOVA showed significant differences with *P* < 0.05. Scale bars: 10 μm in all panels. *ANOVA* analysis of variance, *Clod* clodronate, *PBS* phosphate-buffered saline, *SAH* subarachnoid hemorrhage, *SEM* standard error of the mean

The WT mice were randomly assigned between the following two treatment groups: SAH + ICV PBS and SAH + ICV Clodronate (Clod). Neuronal damage was assessed on POD 7, and cognitive outcome was measured after spatial reversal. We focused on POD 7 because maximal neuronal cell death was reported at that time point [12]. Microglia were depleted in the clodronate-treated group of mice compared with the SAH + PBS group, and neuronal apoptosis was significantly lower in the clodronate SAH group compared with the PBS control group (Student’s *t*-test *P* < 0.05; *n* = 6; Fig. 1a, c). The specificity of clodronate in depleting microglia was evaluated by staining with CD45, a pan-leukocyte marker [16, 17]. Clodronate does not have a significant effect on nonmicroglial cells (Fig. 1b). Cognitive function was assessed by using the Barnes maze, based on our previous work [10]. Differences in cognitive functions were observed between these treatment groups on the days after spatial reversal. The SAH + Clod treatment group after SAH resulted in improved cognitive function when compared with the SAH + PBS group (Student’s *t*-test *P* < 0.05; *n* = 6; Fig. 2).

### Microglial TLR4 is Critical for Brain Damage and Cognitive Function After SAH

**Fig. 3:**
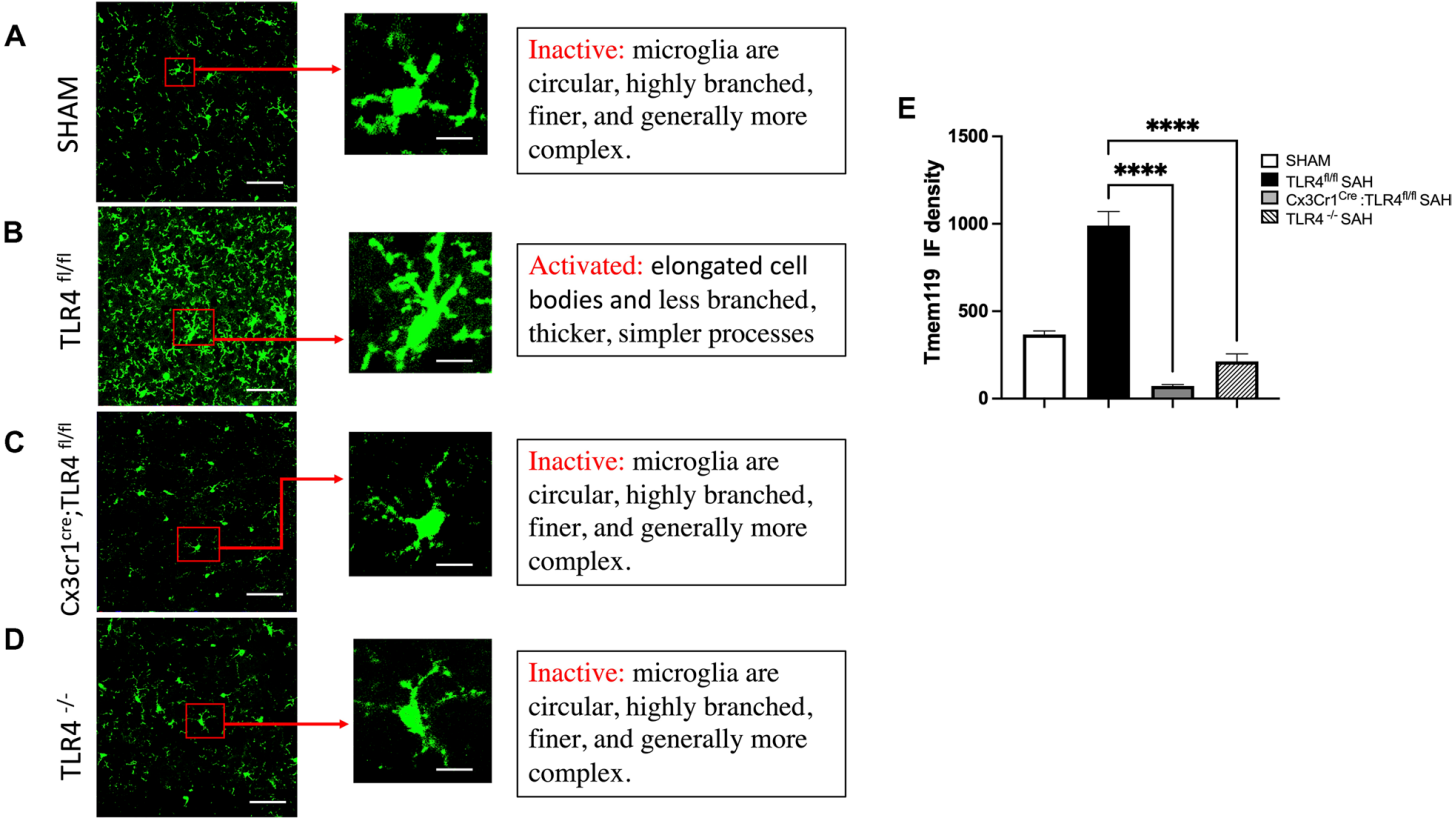
Morphologic activation of microglia and quantification of this activation. Immunohistochemical staining for Tmem-119 (green) showing the morphology of microglial cells after SAH on POD 7 murine brains of sham (**a**), TLR4^fl/fl^ (**b**), Cx3Cr1^Cre^:TLR4^fl/fl^ (**c**), and TLR4 global knockout groups (**d**). Higher magnification images (× 40) show the representative changes in microglial morphology. Microglia with elongated cell bodies and thickened processes indicative of activation. **e** Microglial IF density quantification was performed by measuring the green signal using the ImageJ software. During microglial swelling on activation, the processes remain tend to be thicker than the inactive counterparts. *n* = 6 for each group, and all bar graphs represent mean ± SEM; one-way ANOVA showed significant differences with *P* < 0.05, Scale bar: 50 μm for the low-magnification images and 10 μm for the single-cell images in all panels. *ANOVA* analysis of variance, *ICV* intracerebroventricular, *IF* immunofluorescence, *POD* postoperative day, *SAH* subarachnoid hemorrhage, *SEM* standard error of the mean, *TLR4* toll-like receptor 4

**Fig. 4:**
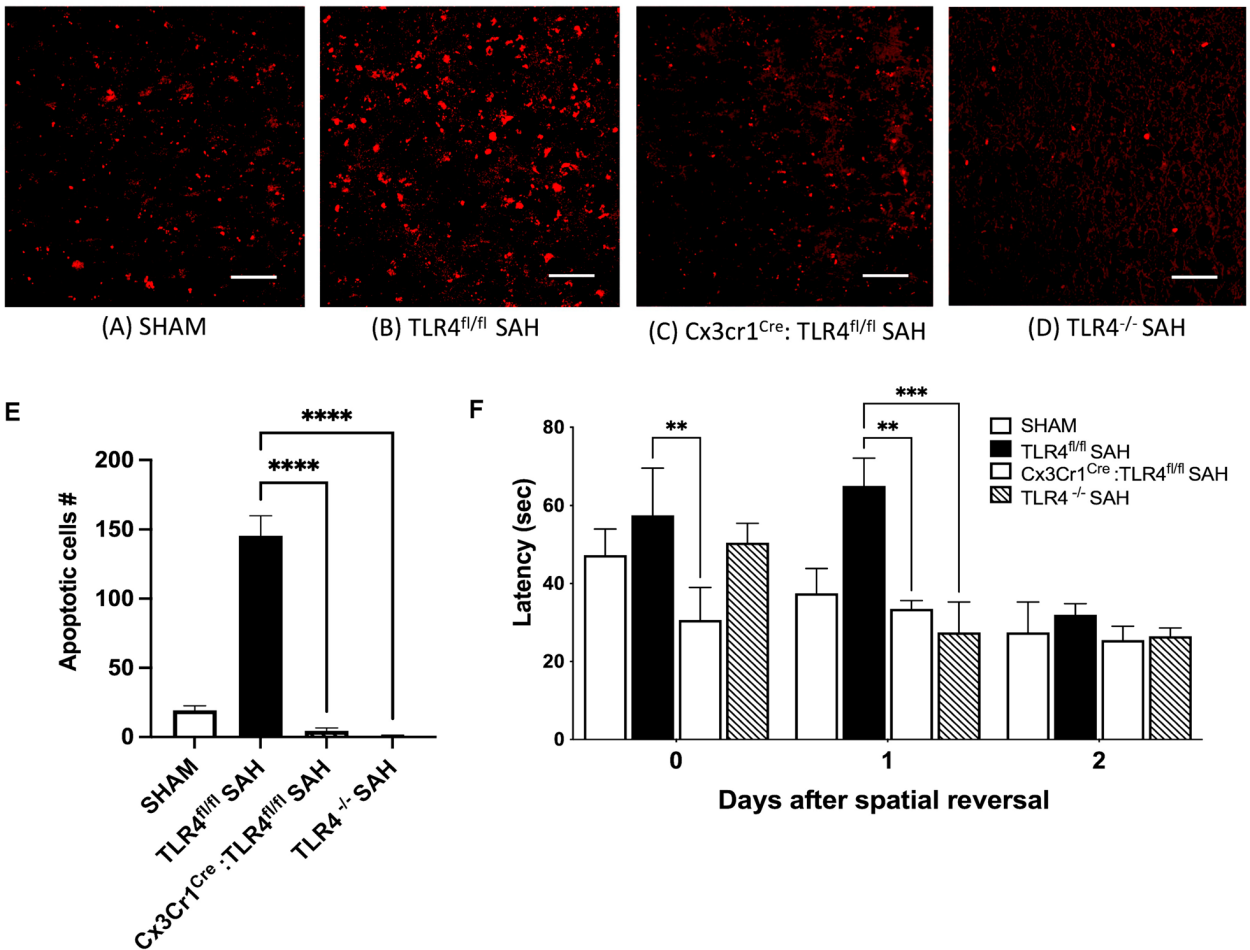
Neuronal apoptosis in subarachnoid hemorrhage model on POD 7 murine brains of sham (**a**), TLR4^fl/fl^ (**b**), Cx3cr1^Cre^:TLR4^fl/fl^ (**c**), and TLR4 (**d**) global knockout groups. Scale bar: 10 μm. **e** Quantification of neuronal apoptosis. *n* = 6 for each group, and all bar graphs represent mean ± SEM; one-way ANOVA *P < 0.05. Scale bars: 10 μm in all panels. **f** Cognitive function test. Total latency in seconds for spatial memory function testing by Barnes maze test for all four groups (*n* = 6 for each group and all bar graphs represent mean ± SEM; two-way ANOVA; *P < 0.05). *ANOVA* analysis of variance, *POD* postoperative day, *SAH* subarachnoid hemorrhage, *SEM* standard error of the mean, *TLR4* toll-like receptor 4

We next sought to determine whether microglial TLR4 was necessary for cell damage and cognitive function after SAH. We analyzed morphological changes to microglia, on the basis of TLR4 expression, by immunostaining for Tmem-119 (Fig. 3a–d). The microglia of the TLR4^fl/fl^ SAH group showed a more activated morphology with elongated cell bodies and thicker processes, which were measured by microglial IF density quantification. There were also more microglia in the TLR4^fl/fl^ SAH group than the other groups (Fig. 3b). Microglia in the Cx3Cr1^Cre^: TLR4^fl/fl^ SAH and TLR4^−/−^ SAH group had microglia with a similar morphology to sham (Fig. 3c, d; one-way ANOVA, *P* < 0.05 vs. sham; *n* = 6; Fig. 3e). To determine the neuronal apoptosis, we performed TUNEL counterstained with neuronal marker, β-III Tubulin in these four groups (Fig. 4a–d). Our data showed there is a significant increase in neuronal damage in TLR4^fl/fl^ SAH group compared with the sham group and other TLR4 deficient groups (one-way ANOVA, *P* < 0.05; *P* < 0.05 vs. sham; *n* = 6; Fig. 4e), which corresponded with significantly impaired cognitive function in the Barnes maze test and an improved cognitive performance in the microglial and global TLR4 knockout mice (Fig. 4f). Again, no difference in task learning was observed prior to SAH.

### TLR4 in Other Glia Do Not Participate in Brain Damage and Cognitive Dysfunction After SAH

**Fig. 5:**
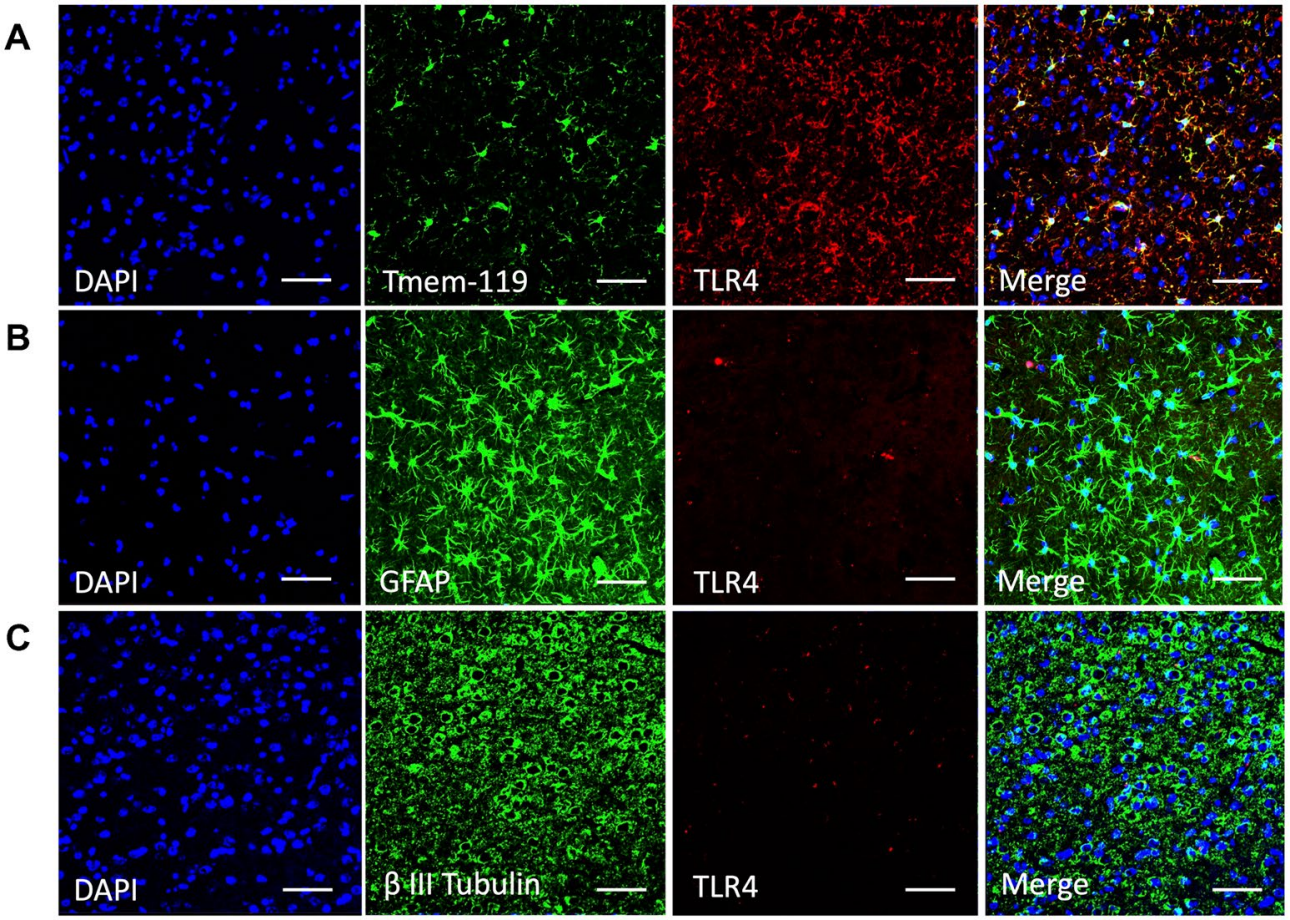
Immunohistochemistry of TLR4 colocalization among the different cell types of the Nestin^Cre^: TLR4^fl/fl^ mice brain. Representative images from four mice per group and four different fields of view on POD 7. **a** The immunohistochemistry across shows staining with DAPI in the first panel, Tmem-119 (for microglia) in the second panel, Toll-like receptor 4 (TLR4) in the third panel, and the merge of panels in the fourth panel. **b** Immunohistochemistry showing individual panels and the merging of panels for astrocytes with TLR4. **c** Immunohistochemistry for β III Tubulin reflecting colocalization of TLR4 and neurons. Scale Bars: 10 μm. *DAPI* 4′,6-diamidino-2-phenylindole, *POD* postoperative day

**Fig. 6:**
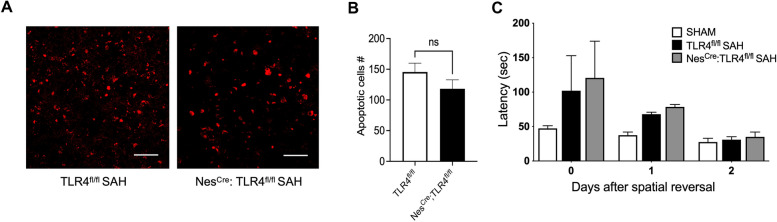
Neuronal apoptosis in SAH model at POD 7 in murine brains. **a** TLR4^fl/fl^ versus Nestin^Cre^:TLR4^fl/fl^. Scale Bar: 10 μm. **b** Quantification of apoptotic cells. **c** Cognitive function test. Total latency in seconds for spatial memory function testing by Barnes maze test for TLR4^fl/fl^ versus Nestin^Cre^:TLR4^fl/fl^ (*n* = 6 for each group and all bar graphs represent mean ± SEM; two-way ANOVA; *P < 0.05). *ANOVA* analysis of variance, *ICV* intracerebroventricular, *POD* postoperative day, *ROI* region of interest, *SAH* subarachnoid hemorrhage, *SEM* standard error of the mean

To determine whether microglial TLR4 is sufficient for neuronal damage rather than other glial TLR4, we performed SAH on Nes^Cre^: TLR4^fl/fl^ mice lacking astrocyte and neuronal TLR4. To confirm the TLR4 expression, the brains were counterstained with DAPI/Tmem-119/TLR4, DAPI/GFAP/TLR4, and DAPI/β-III Tubulin /TLR4. Our data confirmed that both astrocytes and neurons did not express TLR4 (Fig. 5b, c), whereas the microglial colocalization with TLR4 was similar to WT (Fig. 5a). TUNEL data demonstrated a similar level of neuronal damage in the Nes^Cre^: TLR4^fl/fl^ SAH mice compared with the TLR4^fl/fl^ SAH mice (Fig. 6a; one-way ANOVA, *P* < 0.05; *P* < 0.05 vs. sham; *n* = 6; Fig. 6b). No difference was observed in cognitive function, as assessed by the Barnes maze, between the Nes^Cre^: TLR4^fl/fl^ SAH mice and TLR4^fl/fl^ SAH mice (Fig. 6c).

## Discussion

Microglial activation and proliferation after hemorrhagic stroke have been studied with varying results depending on the model used [18–20]. Clodronate is effective at depleting microglia and likely also at depleting border-associated macrophages in neurological disease conditions [21, 22]. We found that SAH inflicts an intracerebral migration of microglia and leads to a secondary neuronal injury that contributes to neuronal cell death and was significantly reduced after microglial depletion using clodronate (Fig. 1a, c, d); however, even though we did not see a significant reduction in the pan-leukocyte CD45 staining (Fig. 1b), it is possible that clodronate treatment may deplete a small, subpopulation of leukocytes not visible on gross immunohistochemical staining. This is a limitation of our study. Others have shown that depletion of phagosomes after SAH was effective at decreasing neuronal apoptosis [12, 22, 23]. In this study, we went one step further to translate the decrease in neuronal apoptosis to a functional outcome and showed that cognitive performance on the Barnes maze was also improved with microglia and phagosomes (Fig. 2).

In neonatal stroke and neurodegenerative diseases, microglia also have a dichotomous role [18, 24, 25]. Regarding SAH, our lab has previously shown a beneficial role with respect to microglial heme-oxygenase-1 and a detrimental role of TLR4 using whole-body knockouts [5, 8], but cell-specific TLR4 knockouts using individual glial populations have never been investigated. We used Cx3Cr1^CreER^:TLR4^fl/fl^ (tamoxifen-inducible to create a microglial specific knockout of TLR4) and Nes^Cre^: TLR4^fl/fl^ (a neuron and astrocyte-specific knockout of the TLR4) in our current study [13]. Although *CX3CR1* is widely expressed on circulating monocytes and central monocytes including microglia, the turnover of microglia is on the order of months, compared with all other leukocytes with *CX3CR1* expression. For this reason, we injected our mice with tamoxifen 1 month before the planned experiments, yielding selective microglial knockouts, as previously described [26, 27]. These TLR4-conditional knockouts on microglia, astrocytes, and neurons demonstrate the role of TLR4 in post-SAH microglial activation, neuronal damage, and cognitive behavior (Figs. 3, 4, 5, 6).

Morphological change is one of the important characteristics of microglial activation. Therefore, in our study we analyzed morphological patterns of microglia immunostaining with Tmem-119. We found that microglia from TLR^fl/fl^ SAH mice showed an increased microglial IF density manifested by elongated cell bodies and thickened processes, likely indicating activation, which significantly decreased in conditional and global knockout of TLR4 in SAH mice (Fig. 3a–e). We also found a significant reduction of neuronal death in the knockouts compared with the TLR ^fl/fl^ SAH group, possibly because of microglial inactivation due to TLR4 deficiency (Fig. 4a–e). A significant improvement of cognitive outcome was also observed in the TLR4 deficient mice (Fig. 4f). These findings demonstrate the involvement of TLR4 in microglial activation as well as in neuronal injury.

TLR4 in an SAH mouse model is largely expressed on microglia, although it is also expressed to a lesser extent on astrocytes and neurons [12, 28]. In the current study, we determined whether microglial TLR4 was sufficient to produced brain injury and cognitive dysfunction after SAH, which is another unique aspect of this study. SAH in Nes^Cre^: TLR4^fl/fl^ mice lacking astrocytic and neuronal TLR4, validated through immunohistochemistry (Fig. 5b, c), showed no differences in neuronal apoptosis or cognitive dysfunction compared with TLR4^fl/fl^ SAH (Fig. 6a–c). This indicates that astrocyte and neuronal TLR4 are not involved in post-SAH cerebral injury.

Cerebral injury seen after SAH is largely mediated by neuroinflammation secondary to lysed red blood cells releasing proteins such as heme, methemoglobin, and high mobility group box-1, all of which are ligands for TLR4 [29, 30]. Our results suggest that microglia, and specifically microglial TLR4, are crucial for neuronal damage and cognitive dysfunction.

Therapies to treat SAH have long been focused on ameliorating vasospasm, with the assumption that DCI or delayed neurological deterioration have a vascular cause. We believe that our body of work establishes a role for neuroinflammation in neurological injury in which microglial TLR4 could provide a promising target for future therapeutics in SAH, and further studies are required to elucidate the molecular mechanism of TLR4 involved in neuronal impairment.

## References

[1] Bederson JB, Connolly ES, Batjer HH, Dacey RG, Dion JE, Diringer MN (2009). Guidelines for the management of aneurysmal subarachnoid hemorrhage: a statement for healthcare professionals from a special writing group of the Stroke Council, American Heart Association. Stroke.

[2] Etminan N, Chang HS, Hackenberg K, de Rooij NK, Vergouwen MDI, Rinkel GJE (2019). Worldwide incidence of aneurysmal subarachnoid hemorrhage according to region, time period, blood pressure, and smoking prevalence in the population: a systematic review and meta-analysis. JAMA Neurol.

[3] Dankbaar JW, de Rooij NK, Velthuis BK, Frijns CJ, Rinkel GJ, van der Schaaf IC (2009). Diagnosing delayed cerebral ischemia with different CT modalities in patients with subarachnoid hemorrhage with clinical deterioration. Stroke.

[4] Hanafy KA, Stuart RM, Khandji AG, Connolly ES, Badjatia N, Mayer SA (2010). Relationship between brain interstitial fluid tumor necrosis factor-alpha and cerebral vasospasm after aneurysmal subarachnoid hemorrhage. J Clin Neurosci.

[5] LeBlanc RH, Chen R, Selim MH, Hanafy KA (2016). Heme oxygenase-1-mediated neuroprotection in subarachnoid hemorrhage via intracerebroventricular deferoxamine. J Neuroinflamm.

[6] Vergouwen MD, Ilodigwe D, Macdonald RL (2011). Cerebral infarction after subarachnoid hemorrhage contributes to poor outcome by vasospasm-dependent and -independent effects. Stroke.

[7] Figueiredo RT, Fernandez PL, Mourao-Sa DS, Porto BN, Dutra FF, Alves LS (2007). Characterization of heme as activator of toll-like receptor 4. J Biol Chem.

[8] Hedblom A, Hejazi SM, Canesin G, Choudhury R, Hanafy KA, Csizmadia E (2019). Heme detoxification by heme oxygenase-1 reinstates proliferative and immune balances upon genotoxic tissue injury. Cell Death Dis.

[9] Kwon MS, Woo SK, Kurland DB, Yoon SH, Palmer AF, Banerjee U (2015). Methemoglobin is an endogenous toll-like receptor 4 ligand-relevance to subarachnoid hemorrhage. Int J Mol Sci.

[10] Akamatsu Y, Pagan VA, Hanafy KA (2020). The role of TLR4 and HO-1 in neuroinflammation after subarachnoid hemorrhage. J Neurosci Res.

[11] Hu Y, Li C, Wang X, Chen W, Qian Y, Dai X (2021). TREM2, driving the microglial polarization, has a TLR4 sensitivity profile after subarachnoid hemorrhage. Front Cell Dev Biol.

[12] Hanafy KA (2013). The role of microglia and the TLR4 pathway in neuronal apoptosis and vasospasm after subarachnoid hemorrhage. J Neuroinflamm.

[13] Thomas AJ, Ascanio-Cortez L, Gomez S, Salem M, Maragkos G, Hanafy KA (2020). Defining the mechanism of subarachnoid hemorrhage-induced pyrexia. Neurotherapeutics.

[14] Schallner N, Pandit R, LeBlanc R, Thomas AJ, Ogilvy CS, Zuckerbraun BS (2015). Microglia regulate blood clearance in subarachnoid hemorrhage by heme oxygenase-1. J Clin Investig.

[15] Ren W, Markel DC, Schwendener R, Ding Y, Wu B, Wooley PH (2008). Macrophage depletion diminishes implant-wear-induced inflammatory osteolysis in a mouse model. J Biomed Mater Res A.

[16] Brandenburg S, Turkowski K, Mueller A, Radev YT, Seidlitz S, Vajkoczy P (2017). Myeloid cells expressing high level of CD45 are associated with a distinct activated phenotype in glioma. Immunol Res.

[17] Hermiston ML, Xu Z, Weiss A (2003). CD45: a critical regulator of signaling thresholds in immune cells. Annu Rev Immunol.

[18] Lin S, Yin Q, Zhong Q, Lv FL, Zhou Y, Li JQ (2012). Heme activates TLR4-mediated inflammatory injury via MyD88/TRIF signaling pathway in intracerebral hemorrhage. J Neuroinflamm.

[19] Simard JM, Tosun C, Ivanova S, Kurland DB, Hong C, Radecki L (2012). Heparin reduces neuroinflammation and transsynaptic neuronal apoptosis in a model of subarachnoid hemorrhage. Transl Stroke Res.

[20] Heinz R, Brandenburg S, Nieminen-Kelha M, Kremenetskaia I, Boehm-Sturm P, Vajkoczy P (2021). Microglia as target for anti-inflammatory approaches to prevent secondary brain injury after subarachnoid hemorrhage (SAH). J Neuroinflammation.

[21] Mrdjen D, Pavlovic A, Hartmann FJ, Schreiner B, Utz SG, Leung BP (2018). High-dimensional single-cell mapping of central nervous system immune cells reveals distinct myeloid subsets in health, aging, and disease. Immunity.

[22] Schneider UC, Davids AM, Brandenburg S, Muller A, Elke A, Magrini S (2015). Microglia inflict delayed brain injury after subarachnoid hemorrhage. Acta Neuropathol.

[23] Lund H, Pieber M, Harris RA (2017). Lessons learned about neurodegeneration from microglia and monocyte depletion studies. Front Aging Neurosci.

[24] Faustino JV, Wang X, Johnson CE, Klibanov A, Derugin N, Wendland MF (2011). Microglial cells contribute to endogenous brain defenses after acute neonatal focal stroke. J Neurosci.

[25] Hawkes CA, McLaurin J (2009). Selective targeting of perivascular macrophages for clearance of beta-amyloid in cerebral amyloid angiopathy. Proc Natl Acad Sci U S A.

[26] Goldmann T, Wieghofer P, Muller PF, Wolf Y, Varol D, Yona S (2013). A new type of microglia gene targeting shows TAK1 to be pivotal in CNS autoimmune inflammation. Nat Neurosci.

[27] Parkhurst CN, Yang G, Ninan I, Savas JN, Yates JR, Lafaille JJ (2013). Microglia promote learning-dependent synapse formation through brain-derived neurotrophic factor. Cell.

[28] Pascual-Lucas M, Fernandez-Lizarbe S, Montesinos J, Guerri C (2014). LPS or ethanol triggers clathrin- and rafts/caveolae-dependent endocytosis of TLR4 in cortical astrocytes. J Neurochem.

[29] Takizawa K, Osawa H, Kojima A, Abraham SJK, Hosaka S (2017). Cystic adventitial disease of popliteal artery with venous aneurysm of popliteal vein: two-year follow-up after surgery. Case Rep Vasc Med.

[30] Murakami K, Koide M, Dumont TM, Russell SR, Tranmer BI, Wellman GC (2011). Subarachnoid hemorrhage induces gliosis and increased expression of the pro-inflammatory cytokine high mobility group box 1 protein. Transl Stroke Res.

